# Rejuvenating potato growth and yield in challenging semiarid and saline sandy Cholistan: harnessing PGPB-coated N and P application strategies

**DOI:** 10.1186/s12870-024-05056-x

**Published:** 2024-05-09

**Authors:** Muhammad Wasim Haider, Muhammad Nafees, Rashid Iqbal, Sajid Ali, Habat Ullah Asad, Farrukh Azeem, Abdel-Rhman Z. Gaafar, Mohamed S. Elshikh, Humaira Rizwana, Heba H. Elsalahy, Ayman M. S. Elshamly, Kassem A. S. Mohammed

**Affiliations:** 1 Department of Horticultural Sciences, Faculty of Agriculture and Environment, The Islamia University of Bahawalpur, Bahawalpur, 63100 Pakistan; 2 Department of Agronomy, Faculty of Agriculture and Environment, The Islamia University of Bahawalpur Pakistan, Bahawalpur, 63100 Pakistan; 3 Department of Horticulture, Bahauddin Zakariya University, Multan, 60000 Pakistan; 4 Centre for Agriculture and Bioscience International, Rawalpindi, 46300 Pakistan; 5 Agri Development, Fauji Fresh n Freeze Ltd, Gulberg II, Lahore, 48000 Pakistan; 6 Department of Botany and Microbiology, College of Science, King Saud University, Riyadh, 11451 Saudi Arabia; 7 Leibniz Centre for Agricultural Landscape Research (ZALF), 15374 Müncheberg, Germany; 8 Water Studies and Research Complex, National Water Research Center, Cairo, 13411 Egypt; 9 Natural Resources Department, Institute of African and Nile Basin Countries Research and Studies, Aswan University, Sahary, 81528 Egypt

**Keywords:** Alkalinity, Crop yield improvement, Fertilizer application approach, Photosynthesis-related metrics, Plant growth promoting bacteria (PGPB)

## Abstract

**Background:**

Potato serves as a major non-cereal food crop and income source for small-scale growers in Punjab, Pakistan. Unfortunately, improper fertilization practices have led to low crop yields, worsened by challenging environmental conditions and poor groundwater quality in the Cholistan region. To address this, we conducted an experiment to assess the impact of two fertilizer application approaches on potato cv. Barna using plant growth-promoting bacteria (PGPB) coated biofertilizers. The first approach, termed conventional fertilizer application (CFA), involved four split applications of PGPB-coated fertilizers at a rate of 100:75 kg acre^–1^ (N and P). The second, modified fertilizer application (MFA), employed nine split applications at a rate of 80:40 kg acre^–1^.

**Results:**

The MFA approach significantly improved various plant attributes compared to the CFA. This included increased plant height (28%), stem number (45%), leaf count (46%), leaf area index (36%), leaf thickness (three-folds), chlorophyll content (53%), quantum yield of photosystem II (45%), photosynthetically active radiations (56%), electrochromic shift (5.6%), proton flux (24.6%), proton conductivity (71%), linear electron flow (72%), photosynthetic rate (35%), water use efficiency (76%), and substomatal CO_2_ (two-folds), and lowered non-photochemical quenching (56%), non-regulatory energy dissipation (33%), transpiration rate (59%), and stomatal conductance (70%). Additionally, the MFA approach resulted in higher tuber production per plant (21%), average tuber weight (21.9%), tuber diameter (24.5%), total tuber yield (29.1%), marketable yield (22.7%), seed-grade yield (9%), specific gravity (9.6%), and soluble solids (7.1%). It also reduced undesirable factors like goli and downgrade yields by 57.6% and 98.8%, respectively. Furthermore, plants under the MFA approach exhibited enhanced nitrogen (27.8%) and phosphorus uptake (40.6%), with improved N (26.1%) and P uptake efficiency (43.7%) compared to the CFA approach.

**Conclusion:**

The use of PGPB-coated N and P fertilizers with a higher number of splits at a lower rate significantly boosts potato production in the alkaline sandy soils of Cholistan.

## Background

Potato is globally the third-largest food crop consumption-wise and the fourth largest production-wise [1, 2]. It is an excellent source of energy due to its richness in both carbohydrates and minerals [3]. In Pakistan, potato is grown as a main vegetable on a land of 0.78 million acres, with a projected yearly yield of 7.9 million tons, on an average of 10.1 tons acre^–1^ [4]. There is always a gap between actual and potential yields [5]. The “yield potential concept” emphasizes that the potential yield in a natural production system cannot be fully achieved due to biotic or/and abiotic stresses which interfere with the potato crop during plant growth and tuber development. A sufficient supply of nutrients can fortify the potato crop against stressors and assist in achieving high quantitative and qualitative yields [6]. Nevertheless, providing a nutritious food supply for an expanding human population necessitates an efficient nutrient management system. The excessive utilization of chemical fertilizers to increase yields for increased income is damaging to the planet’s resources [7]. Splitting nutrient applications according to a plant’s requirements has now become a common trend; the correct timing of fertilizer application to correspond to a plant’s requirements during the growing period can only give an economical and optimal yield of potato [8]. Sustainable agriculture is also desirous for increased nutrient use efficiency to maintain crop productivity and reduce environmental damage [9].

Nitrogen (N) and phosphorous (P) are the most common essential nutrients fertilized in potato production [10–12]. N is the most limiting nutrient and is required in higher quantities by most plants than any other plant nutrient [13]. So, the rate of N application is critical to optimize the potato tuber yield and quality [14, 15]. Although the effect of N on tuber yield and quality has been well documented [14, 16, 17], the growers, being unconscious about specific plant growth aspects, i.e., nutrient uptake efficiency, apply large quantities of fertilizers to maximize the yield [18]. As a result, our water systems and environment are polluted, in addition to the economic loss suffered by the growers in the form of nutrient waste [11]. A higher N availability has a tremendous effect on vegetative growth and the light interception of a crop, which encourages tuber yield [19]. Lower rates of N not only result in lower yield but also decrease tuber size due to early defoliation. In contrast, lower N may slow photosynthesis and negatively influence the partitioning of photoassimilates from leaves to tubers [20, 21]. Alternatively, excessive N outside the root area of plants may be wasted through leaching or gaseous emissions [22]. Furthermore, the excessive N prompts a dry matter percentage of plant parts other than the tubers [23]. The optimum application rate of N leads to increased total and marketable yields [14, 24], while a deficiency in N leads to carbohydrate accumulation in leaves. As a result, a higher level of carbon is allocated to the root, which hence increases the root: shoot ratio [25, 26]. Splitting N application lowers the risk of its loss, especially on sandy soils [14], as well as meeting the actual demand of potato crops during upcoming developmental stages. However, the number of split applications still needs to be precisely managed, as many factors affect the efficiency of this process.

P is the second most limiting nutrient for potato crop production after N, and its availability is largely influenced by the soil pH. A pH of 6.0-7.5 is supposed to be the best range in terms of P solubility. Under alkaline soil conditions, P uptake is impaired due to the formation of poorly soluble magnesium or calcium precipitates [27]. Plants grown under P deficit soil develop a smaller leaf area, which influences light interception and hence photosynthesis, resulting in a poor crop growth rate [28, 29]. Like N deficiency, P deficiency also instigates increased photoassimilate allocation to the roots [30]. So, similar to N, a plant also requires P during later stages of development, i.e., tuber setting [31, 32]. So, P should also be applied as an in-season fertilizer like N, besides its pre-planting application.

Plant growth-promoting bacteria (PGPB) also have an important role in sustainable agriculture. PGPB can enhance plant growth through the production of phytohormones, enhance symbiotic N_2_ fixation, and enhance phosphorus absorption by inhabiting plant roots (rhizosphere) [33]. Its use as a frontier point could achieve an optimistic effect on plants and reduce the hazardous impact of fertilizers on the environment.

Cholistan is the second-largest desert in Pakistan, and covers a significant piece of arable land (1.8%) in Pakistan but its yield per unit area is quite low. One of the major constraints for low yields in Cholistan region is the accumulated high levels of salts in the soil and water. Moreover, the growers are adopting conventional fertilizer application practices, using higher rates of fertilizers in a total of three to four splits [14]. The use of PGPB-coated N and P fertilizers at appropriate rates and splits would be a promising approach to boost the total and marketable yields in a saline environment. There is very little published research on the effect of application rate and frequency optimization of PGPB enriched fertilizers on the growth, yield, and quality of potato crop. Therefore, investigations on improving the nutrient-uptake efficiency of potato crop in order to improve its yield and quality will be very effective in this region.

## Results

### Temperature, relative humidity, precipitation, and sunshine trend

**Fig. 1:**
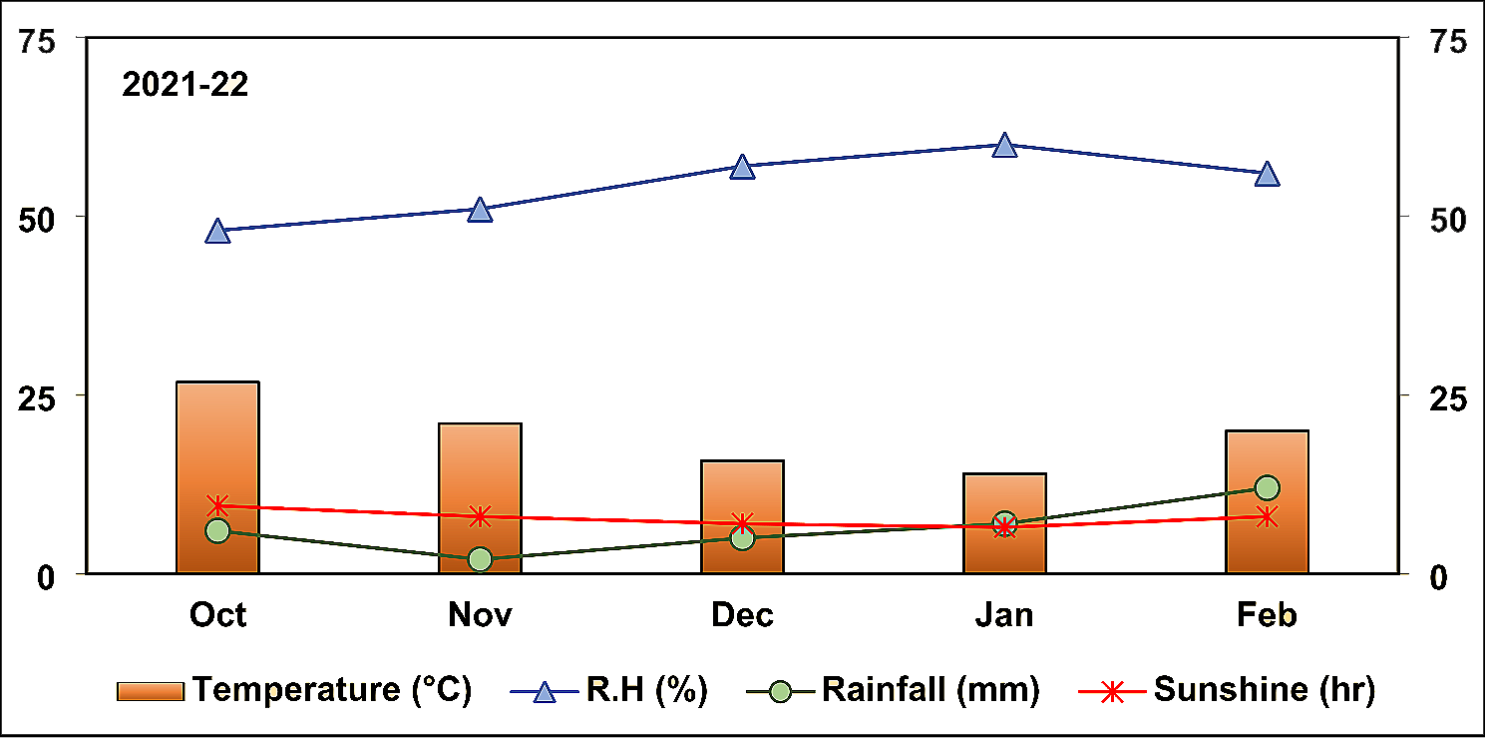
Average values of air temperature, relative humidity, total rainfall, and sunshine for the experimental site during the duration of experiment in 2021-22

The air temperature gradually decreased from the starting month (Oct) (26.8 °C) of the experimental period to the second last month (Jan) (14 °C), but increased during the last month (Feb) (20 °C) (Fig. 1). The relative humidity remained steady throughout the experimental period, with an average minimum and maximum humidity of 48 and 60%, respectively (Fig. 1). The rain shower was comparatively higher (6 mm) at the beginning of the experiment in the month of Oct than in the succeeding month (Nov) (2 mm) (Fig. 1). Later on, the potato crop received rainfall in an increasing pattern (Fig. 1). The sunshine duration also fluctuated over time; it was highest (9.5 h) initially in the month of Oct, thereby decreasing until Jan (6.5 h) and increasing in the month of Feb (8 h) (Fig. 1).

### Physico-chemical analysis of soil and irrigation water

The selected physical and chemical attributes of the top 30 cm of soil are given in Table 1. The soil was medium in alkalinity, with pH values ranging from 8.3 to 8.6 and EC values ranging from 1.80 dS m^–1^ to 2.0 dS m^–1^ (Table 1). The sand contents in the soil ranged from 63 to 65%, and their textural class was sandy loam (Table 1). The cation exchange capacity (CEC) of soil ranged from 6.9 to 8.6 c mol kg^–1^ (Table 1). This might be due to the accumulation of a larger amount of salts (0.71–1.03 g Kg^–1^) in the soils of the study area (Table 1). The soil organic matter ranged from 0.28 to 0.63%. The total nitrogen concentration ranged from 0.041 to 0.050%. The available content of P and K ranged from 0.4 ppm to 0.6 ppm and 55 ppm to 86 ppm, respectively (Table 1). The selected physical and chemical attributes of irrigation water are given in Table 2. The irrigation water was also medium in alkalinity and sodicity hazard (Table 2).

**Table 1:** Physical and chemical analyses of soil of the experimental site prior to planting

Particular	Unit	Values at two different soil depths	
		0–15 cm	16–30 cm
Soil texture	-	Sandy loam	Sandy loam
Sand	%	63	65
Silt	%	22	25
Clay	%	15	10
Saturation	%	30	30
pH	-	8.3	8.6
EC	dS m^–1^	2.00	1.80
Total dissolved salts	ppt	1.03	0.71
Organic matter	%	0.63	0.28
CEC	c mol kg^–1^	8.6	6.9
Total N	%	0.050	0.041
Available P	ppm	0.6	0.4
Available K	ppm	86	55

**Table 2:** Physical and chemical analyses of irrigated water used in the experiment

Particular	Unit	Value
pH	-	7.7
EC	dS m^–1^	1.23
Total dissolved salts	g L^–1^	1.20
Ca + Mg	Meq L^–1^	7.37
Na	Meq L^–1^	4.93
CO_3_	Meq L^–1^	-
HCO_3_	Meq L^–1^	2.68
Cl	Meq L^–1^	1.12
Sodium adsorption ratio	-	2.57
Residual sodium carbonate	Meq L^–1^	0.31

### The growth-related attributes in potato cv. Barna

**Fig. 2:**
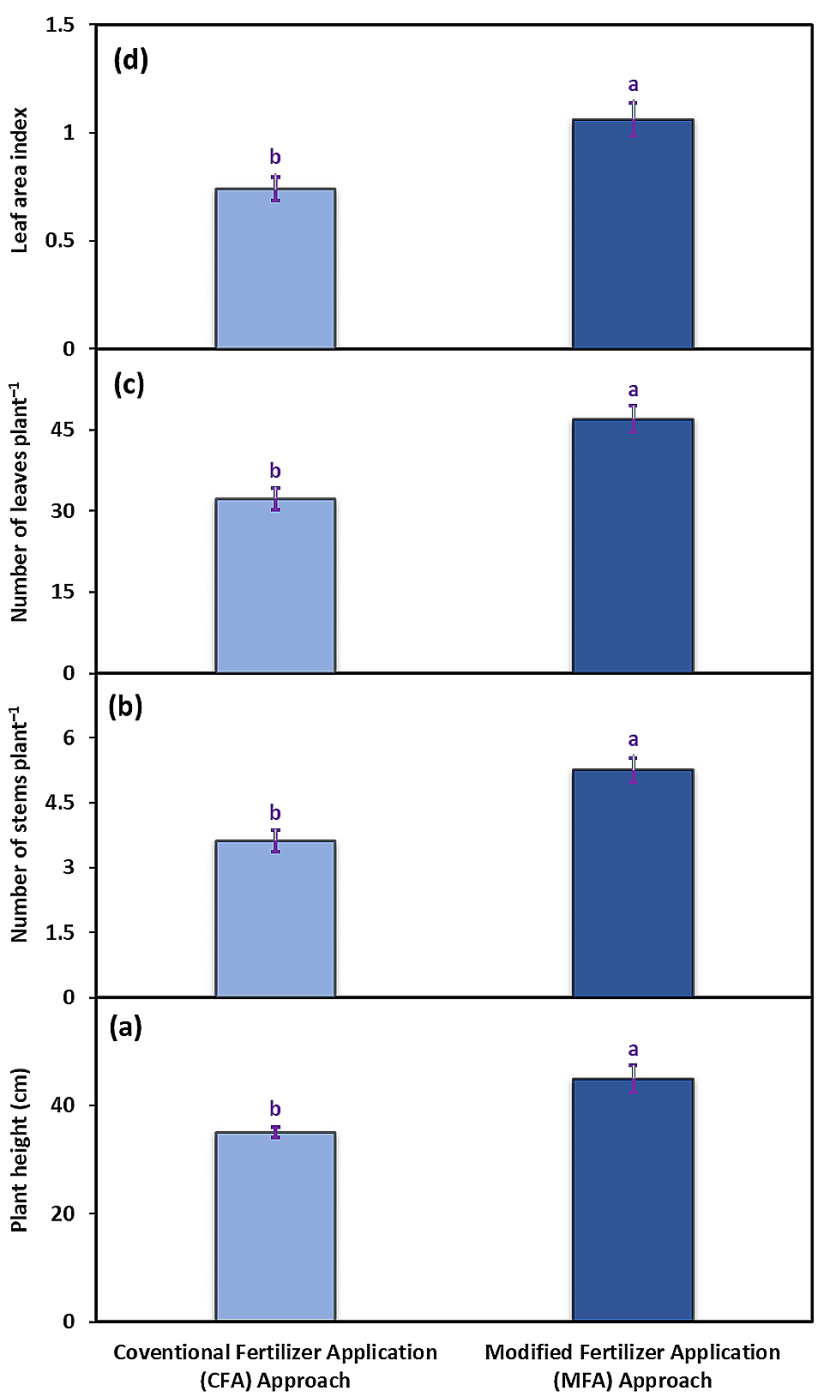
Plant height (**a**), number of stems plant^–1^ (**b**), number of leaves plant^–1^ (**c**), and leaf area index (**d**) of potato plants cv. Barna receiving PGPB coated N and P at the rate 100:75 kg acre^–1^ in four splits (CFA approach) and PGPB coated N and P at the rate 80:40 kg acre^–1^ in nine splits (MFA approach). The bars indicate the standard error (±) of the mean (*n* = 4). Lettering denotes statistical variations between the treatment means carried out using Tukey’s HSD Test at the *P* ≤ 0.05 after analysis of variance

The trial clearly demonstrated that there were significant differences between the conventional fertilizer application (CFA) approach and the modified fertilizer application (MFA) approach of PGPB-coated N and P for plant height, number of stems and leaves plant, and leaf area index of potato cv. Barna. The MFA approach resulted in 28% taller plants compared to the CFA approach (Fig. 2a). The plants grown under the MFA approach also produced a 45% greater number of stems than those grown under the CFA approach (Fig. 2b). The number of leaves was about 46% higher in the plants grown under the MFA approach than those grown under the CFA approach (Fig. 2c). Similarly, leaf area index was about 36% greater in the plants grown under the MFA approach compared to those grown under the CFA approach (Fig. 2d). Overall, an upsurge in the growth attributes was observed when the rate of fertilizers application was lowered but their number increased (Fig. 2).

### The fluorescence-related attributes in potato cv. Barna

**Fig. 3:**
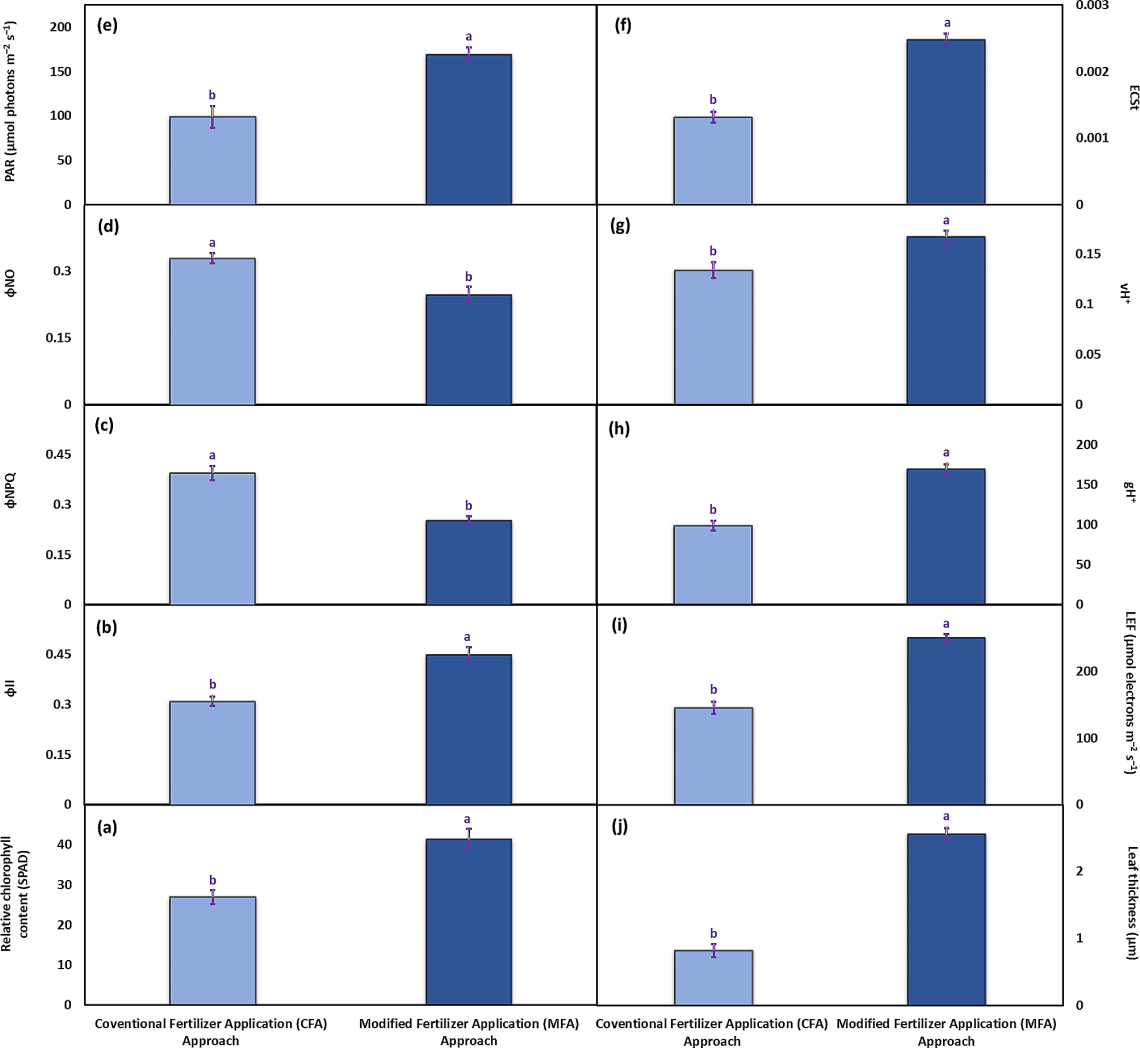
The fluorescence-related attributes i.e., relative chlorophyll content (**a**), quantum yield of photosystem II (ф_II_) (**b**), non-photochemical quenching (ф_NPQ_) (**c**), non-regulatory energy dissipation (ф_NO_) (**d**), photosynthetically active radiation (PAR) (**e**), magnitude of electrochromic shift (ECSt) (**f**), proton flux (vH^+^) (**g**), proton conductivity (gH^+^) (**h**), linear electron flow (LEF) (**i**), and leaf thickness (**j**) recorded in potato plants cv. Barna receiving PGPB coated N and P at the rate 100:75 kg acre^–1^ in four splits (CFA approach) and PGPB coated N and P at the rate 80:40 kg acre^–1^ in nine splits (MFA approach). The bars indicate the standard error (±) of the mean (*n* = 4). Lettering denotes statistical variations between the treatment means carried out using Tukey’s HSD Test at the *P* ≤ 0.05 after analysis of variance

The CFA and MFA approaches showed significant differences for all the studied fluorescence-related attributes. The plants receiving N and P under the CFA approach had a 53% rise in relative chlorophyll content compared to those receiving N and P under the MFA approach (Fig. 3a). The quantum yield of photosystem II (Φ_II_) was 45% higher in the plants grown under the CFA approach than those grown under the MFA approach (Fig. 3b). Non-photochemical quenching (Φ_NPQ_) was noted to be around 56% lower in the plants grown under the MFA approach than those grown under the CFA approach (Fig. 3c). The non-regulatory energy dissipation (Φ_NO_) was also found to be 33% lower in the plants grown under the MFA approach than those grown under the CFA approach (Fig. 3d). The values of photosynthetically active radiation (PAR) were detected to be comparably 56% greater in the plants grown under the MFA approach compared to the CFA approach (Fig. 3e). However, the plants grown under the MFA approach produced only 5.6% stronger electrochromic shift (ECSt) than the CFA approach (Fig. 3f). For proton flux (vH^+^), a 24.6% increase was recorded in the plants grown under the MFA approach than CFA (Fig. 3g). In the case of proton conductivity (gH^+^), the plants under MFA approach exhibited approximately 71% higher values than those under CFA approach (Fig. 3h). In terms of linear electron flow (LEF), the plants under MFA approach displayed about a 72% increase compared to the CFA (Fig. 3i). Regarding leaf thickness, the MFA approach showed a three-fold increase compared to the CFA approach (Fig. 3j).

### The gas exchange-related attributes in potato cv. Barna

**Fig. 4:**
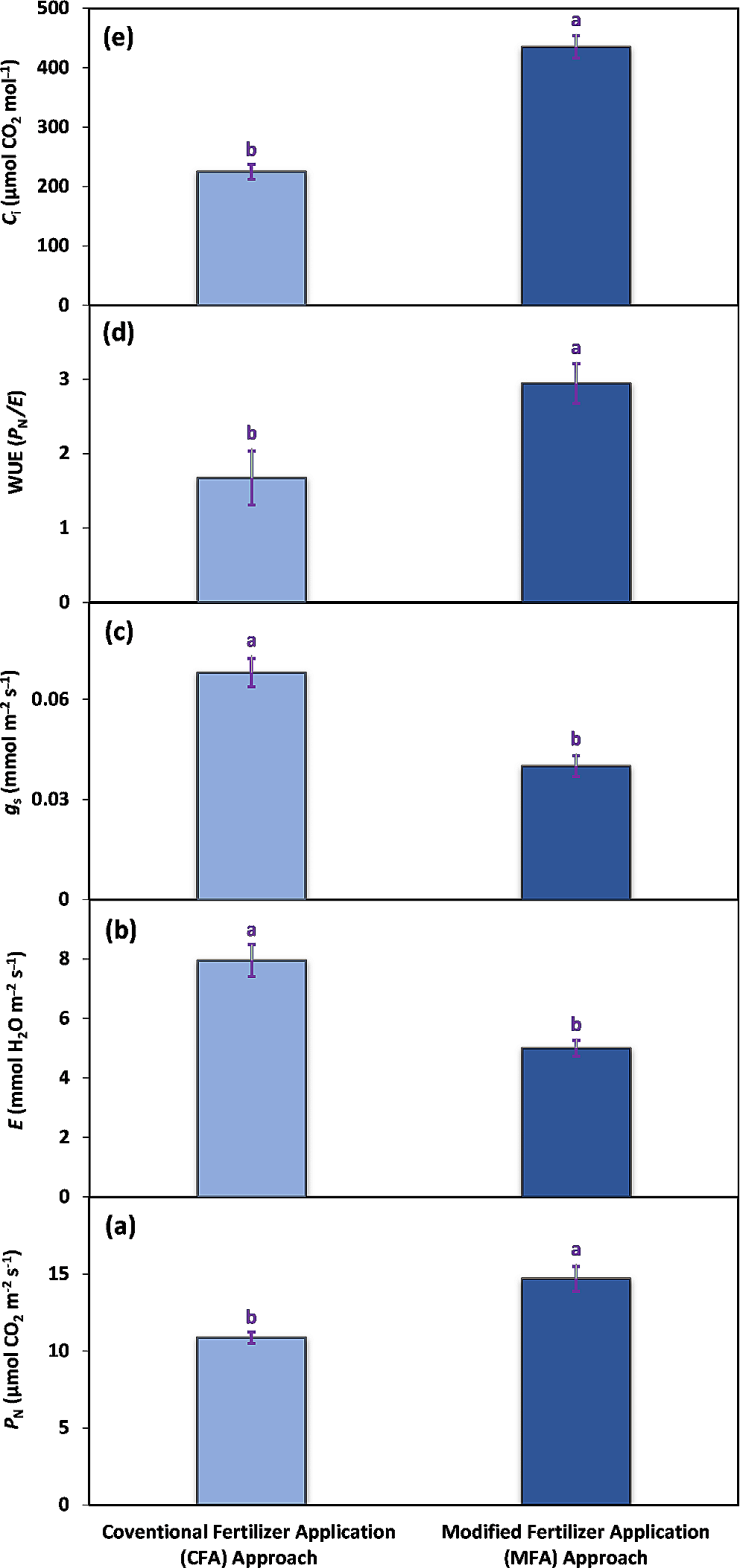
The gas exchange-related attributes i.e., photosynthetic rate (*P*_N_) (**a**), transpiration rate (*E*) (**b**), stomatal conductance (*g*_s_) (**c**), water use efficiency (WUE) (**d**), and substomatal CO_2_ (*C*_i_) (**e**) recorded in potato plants cv. Barna receiving PGPB coated N and P at the rate 100:75 kg acre^–1^ in four splits (CFA approach) and PGPB coated N and P at the rate 80:40 kg acre^–1^ in nine splits (MFA approach). The bars indicate the standard error (±) of the mean (*n* = 4). Lettering denotes statistical variations between the treatment means carried out using Tukey’s HSD Test at the *P* ≤ 0.05 after analysis of variance

A significant effect on gas exchange-related attributes was recorded under two different fertilizer application approaches. Under the MFA approach, the photosynthetic rate of potato plants was about 35% higher than that under CFA approach (Fig. 4a). With the MFA approach, the transpiration rate was also lower (5 mmol H_2_O m^–2^ s^–1^), approximately a 59% decrease from the CFA approach (7.9 mmol H_2_O m^–2^ s^–1^) was noticed (Fig. 4b). Furthermore, the application of the modified fertilizer approach resulted in a decreased stomatal conductance (0.040 mmol m^–2^ s^–1^) of plants, comparably 70% less than the CFA approach (0.068 mmol m^–2^ s^–1^) (Fig. 4c). Following the MFA approach, the water use efficiency (WUE) increased from 1.67 to 2.94, reflecting a significant 76% rise compared to the CFA approach (Fig. 4d). In the end, the substomatal CO_2_ level was also found to be higher (435 µmol CO_2_ mol^–1^) in the plants grown under MFA approach rather than CFA (225 µmol CO_2_ mol^–1^) indicating a two-fold comparable increase (Fig. 4e).

### The yield-related attributes in potato cv. Barna

**Fig. 5:**
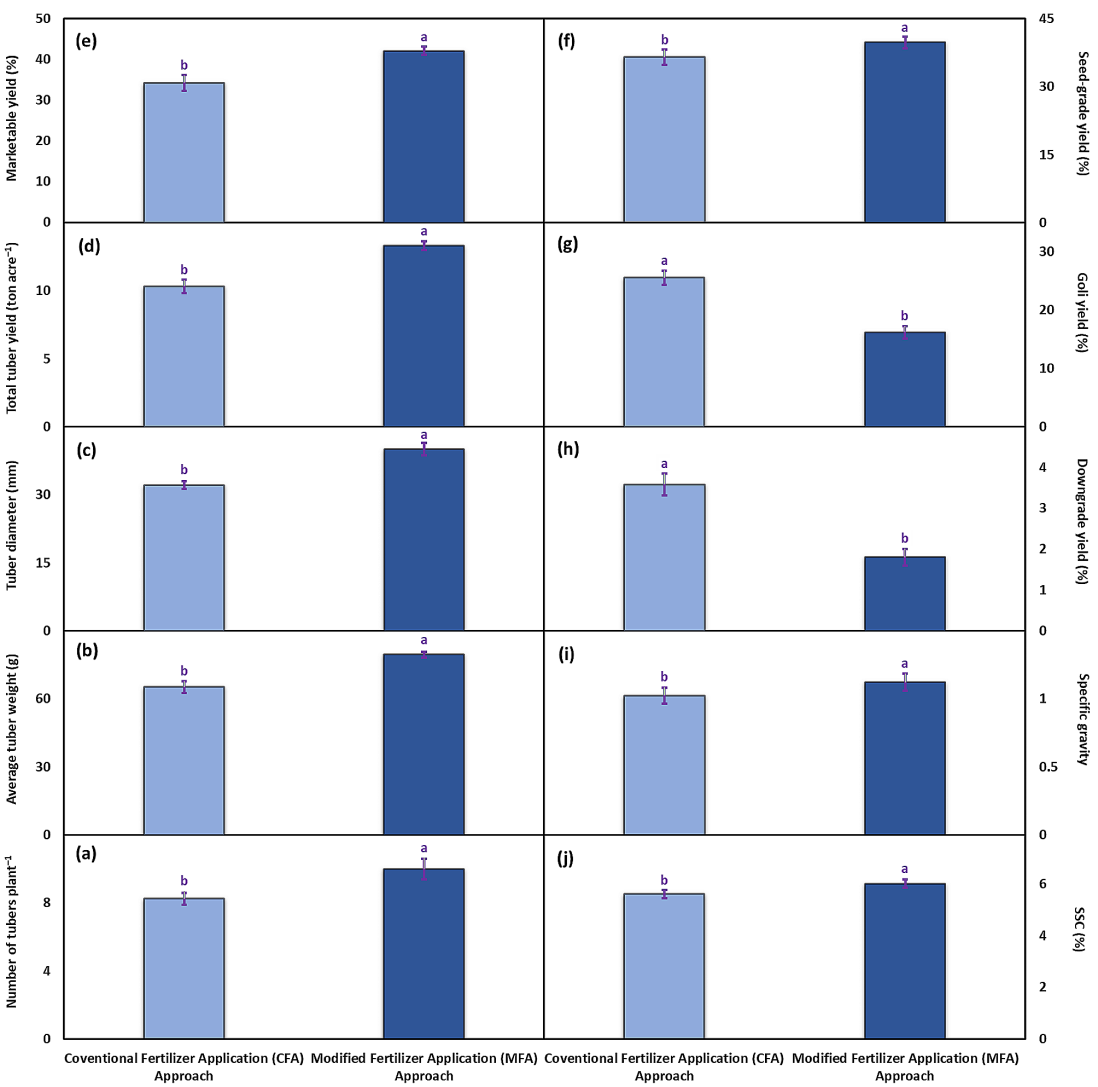
The tuber yield-related attributes i.e., number of tubers plant^–1^ (**a**), average tuber weight (**b**), tuber diameter (**c**), total tuber yield (**d**), marketable yield (**e**), seed-grade yield (**f**), goli yield (**g**), downgrade yield (**h**), specific gravity (**i**), and SSC (**j**) recorded in potato cv. Barna receiving PGPB coated N and P at the rate 100:75 kg acre^–1^ in four splits (CFA approach) and PGPB coated N and P at the rate 80:40 kg acre^–1^ in nine splits (MFA approach). The bars indicate the standard error (±) of the mean (*n* = 4). Lettering denotes statistical variations between the treatment means carried out using Tukey’s HSD Test at the *P* ≤ 0.05 after analysis of variance

Significant variations were observed between MFA and CFA approaches for number of tubers plant^–1^, average tuber weight, tuber diameter, total tuber yield, marketable yield, seed-grade yield, goli yield, downgrade yield, specific gravity, and soluble solid content. The number of tubers plant^–1^ increased from 8 to 10 upon the application of the modified fertilizer approach, resulting in a 21% increase from the CFA approach (Fig. 5a). Moreover, the average tuber weight also improved from 65.2 g to 79.5 g, representing a 21.9% increment over the CFA approach (Fig. 5b). Under the MFA approach, the tuber diameter was noticeably greater (32.2 mm) than the CFA approach (40.1 mm), representing a 24.5% comparable increase (Fig. 5c). The total tuber yield was determined to be 10.3 kg acre^–1^ under CFA approach, while this climbed to 13.3 kg acre^–1^ under MFA approach, showcasing about a 29.1% comparable increase (Fig. 5d). Regarding the marketable yield, the MFA approach showed a 22.7% improvement over the CFA approach (Fig. 5e). Similarly, in the case of seed-grade yield, the MFA approach performed 9% better compared to the CFA approach (Fig. 5f). Alternatively, the goli and downgrade yield percentages were found to be 57.6% and 98.8% lower under the MFA approach than CFA (Fig. 5g-h). The specific gravity and soluble solid content were about 9.6% and 7.1% higher in the plants grown under the MFA approach compared to CFA (Fig. 5i-j).

### The nutrient uptake in potato cv. Barna

**Fig. 6:**
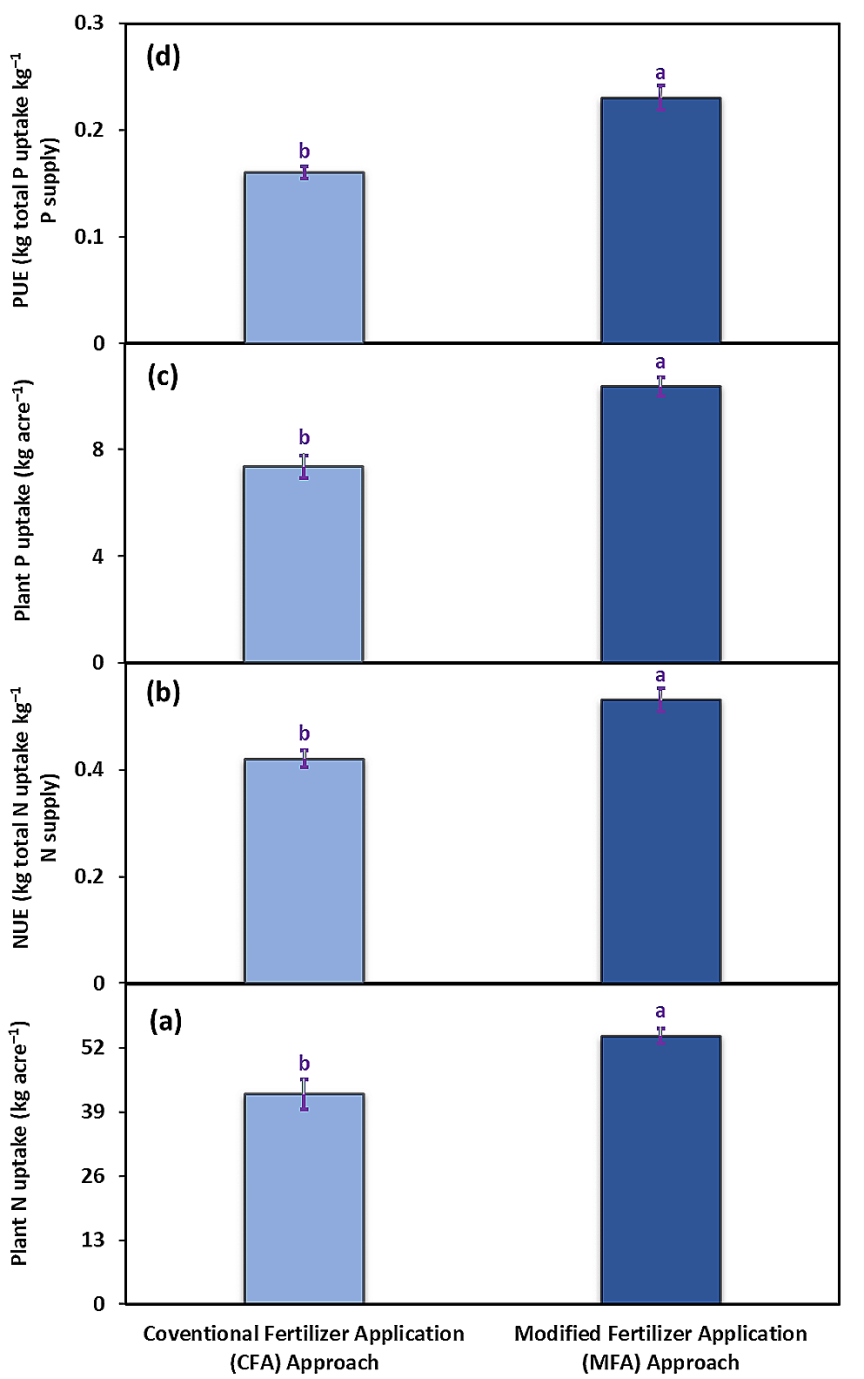
Plant N uptake (**a**), nitrogen uptake efficiency (NUE) (**b**), plant P uptake (**c**), and P uptake efficiency (**d**) assessed in potato cv. Barna receiving PGPB coated N and P at the rate 100:75 kg acre^–1^ in four splits (CFA approach) and PGPB coated N and P at the rate 80:40 kg acre^–1^ in nine splits (MFA approach). The bars indicate the standard error (±) of the mean (*n* = 4). Lettering denotes statistical variations between the treatment means carried out using Tukey’s HSD Test at the *P* ≤ 0.05 after analysis of variance

Total plant N uptake, nitrogen uptake efficiency, total plant P uptake, and P uptake efficiency were significantly affected by both MFA and CFA approaches. Total plant N uptake increased by 27.8% after the application of the modified fertilizer approach, compared to the CFA approach (Fig. 6a); as the nitrogen uptake efficiency improved by almost 26.1% in the plants grown under the MFA approach than those under the CFA approach (Fig. 6b). In spite of this, the MFA approach also enhanced the total P uptake of experimental potato plants by 40.6% compared to the CFA approach (Fig. 6c) by enhancing their P uptake efficiency by almost 43.7% compared to those grown under the CFA approach (Fig. 6d).

## Discussion

The vegetative growth requirements of ‘Barna’ were related to the appropriate N and P supply. As the right amount of N and P fertilization at the early stage of crop favors stem growth by enhancing carbohydrate production. In this trial, the CFA approach produced fewer stem plant^− 1^, while an increase in stem numbers was observed with an increase in the number of split applications in the MFA approach, which indicates that an adequate supply of N and P is essential for maximum stem production. This might be due to the positive impact of a regular supply of N and P, as supported by Wubengeda et al. [34], Najm et al. [35], and Al-Moshileh et al. [36], who reported that balanced fertilization produced the maximum number of stems and leaves plant^− 1^. In the CFA approach, 50% of the total N and P application was made just prior to planting. In the first 2–4 weeks after planting, most of the nutrition needs of the plant are provided by the seed tuber. Thus, little of the applied N and P is expected to be taken up by the plant. Since October, 2021 was a relatively wet month in this study, likely much of the early application of N and P would have been lost to leeching, particularly on the sandy loam soils of this study. A more balanced approach to fertilization, such as in the MFA approach, likely not only supplied critical N and P when the plant most needed them but also applied N and P at times when rainfall was less, and hence, leaching was likely less. The results are also in agreement with the findings of Ekin [7], who observed a two-fold increase in the stem number of potato var. Caspar with PGPB (*Bacillus subtilis*) inoculation compared to the control. Also, N and P have a decisive impact on plant height, as plants under the MFA approach were 12% taller than those under the CFA approach. Ekin [7], Firew et al. [37], Israel et al. [38], and Ayichew et al. [39] have all noted a similar trend of N and P impact on plant height. Overall, split application of PGPB fortified N and P fertilizers maximally improved the vegetative growth of potatoes in this study.

The chlorophyll contents reflect the plant health and also serve as an indicator to assess the functionality of photosynthetic appartus. The results of the current study revealed that with increasing frequency of N from four to nine splits, relative chlorophyll contents of potato leaves increased by 5.2%. Similarly, Mauromicale et al. [40] also reported an increase in chlorophyll contents due to the appropriate rate of N. Our findings also support the results of previous study carried out by Morais et al. [41] in which plants of strawberry cv. Camarosa, in the presence of *Pedobacter spp.*, were found to have a 4.1% greater chlorophyll contents compared to non-inoculated. The fluctuations in chlorophyll fluorescence induced by alterations in N and P application rates and frequencies may be attributed to a direct response in chlorophyll content. In our current investigation, two different rates and frequencies of N and P application have led to a statistically significant reduction (*P* ≤ 0.05) in photosystem II efficiency. This is strongly validated by our findings, as linear electron flow reduced with increasing the frequency and decreasing the application rate exhibiting a strong relationship with chlorophyll levels. According to Kleinwächter and Selmar [42], an improvement in leaf chlorophyll content and photosystem-II efficiency is generally associated with an improved electron transport to the photosystem-II electron acceptor. Previously, in studies on *Solanum melongena* [43] and *Ricinus communis* [44], a decline in the effectiveness of photosystem-II and an increase in non-photochemical quenching have been observed as a response to heat and salt stresses, respectively. The findings showed that MFA approach had a significant positive impact on both the relative conductivity of the thylakoid membrane to protons and the proton conductivity of chloroplast ATP synthase. It is crucial to know that the thylakoid membrane plays a vital role in the infrastructure of the photosynthetic light reaction. In this study, proton flux and proton conductivity were both found stronger under MFA approach. This can be related to the findings of Avenson et al. [45], who discovered that photosystem II, ATP synthase, and electron transfer are all located in the thylakoid membrane and are positively correlated. The findings of this study also indicate that increasing the frequency with lower nutrient application rate has the potential to enhance the integrity of the thylakoid membrane structure and preserve the stability of membrane’s permeability, supported by Sailaja et al. [46] and Kalaji et al. [47], who noted that it is important to maintain the stabilility of thylakoid memebrane for the process of photosynthesis.

The optimum frequency of N and P application improves the gas-exchange attributes of potato plants to increase food production in the form of storage tissues i.e. tubers. The plants grown under MFA approach had a higher rate of photosynthesis and water use efficiency compared to both treatments while lowest transpiration rate and stomatal conductance. Previously, Mathur et al. [48] and Canellas et al. [49] also reported improvement in gaseous exchanges of the maize plants, applied fertilizers in splits along with bacterial strains. An increase in photosynthetic capacity by PGPB has also been observed in other plant species, such as wheat [50], sugar beet [51], and pepper [52]. Due to this applications in splits of PGPB coated nutrients, a substantial difference in the growth and gas-exchange of plants may have occurred.

**Fig. 7:**
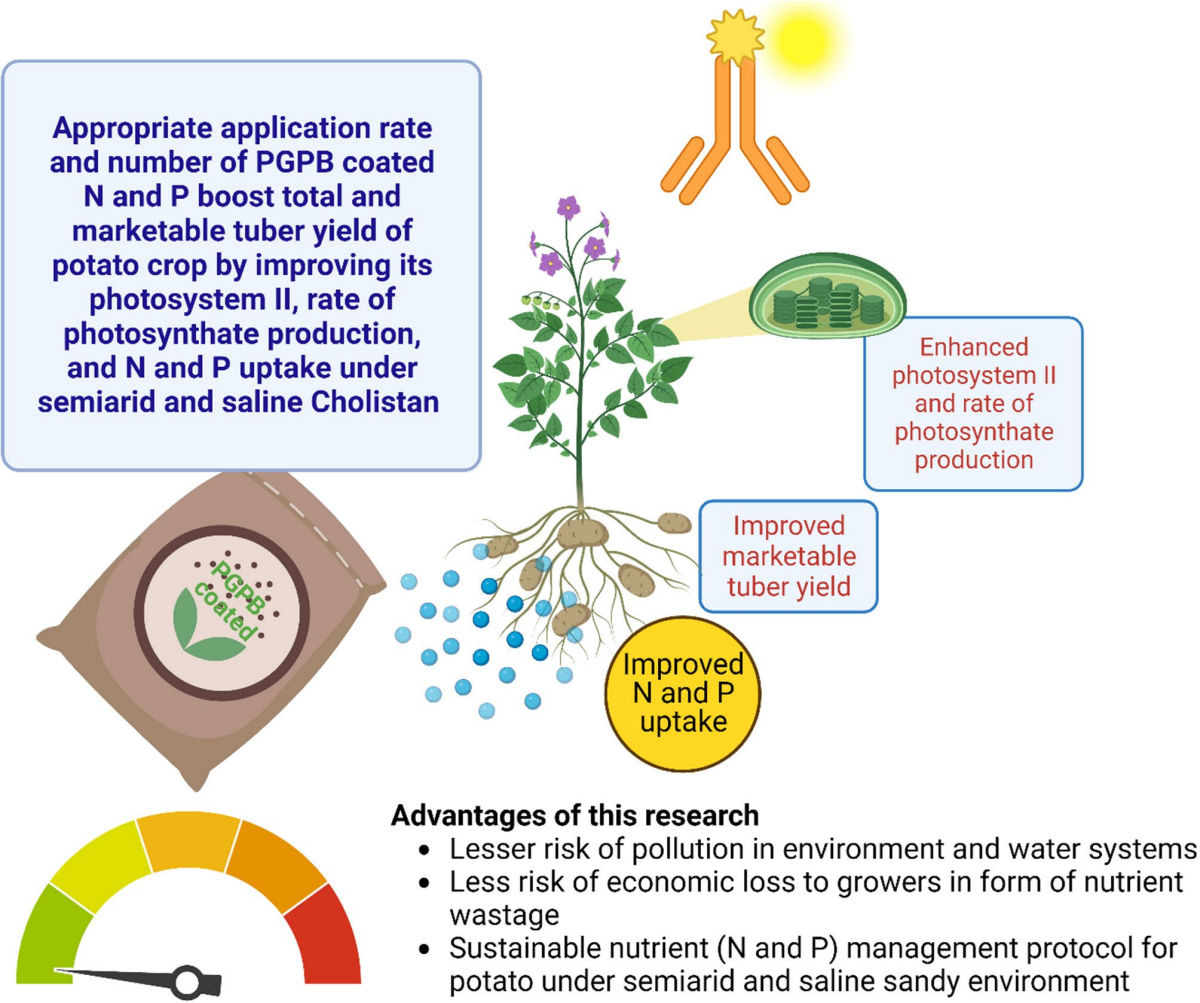
A pictorial illustration of the impact and advantages of adopting the modified fertilizer application (MFA) approach in potatoes

According to De la Morena et al. [53], potato yield is based upon ‘stems numbers per plant’, and ‘average tuber weight’. Both parameters in this study were greatly influenced by the number of split applications of N and P fertilizers. The plants in this research with a greater number of stems and average tuber weight attained greater total and rashan-grade yields. The increased vegetative growth particularly increases in the number of stems and LAI due to improved rate of photosynthesis promoted tuber bulking that resulted in increased tuber weight. Firew et al. [37], Israel et al. [38], and Casa et al. [54] also found a decrease in tuber weight with the reduction in frequency of N and P fertilizer. However, the plants under MFA approach had maximum rates of photosynthates production and so attained greatest tuber weight and so the maximum proportion of marketable tubers (Fig. 7). Canellas et al. [49] and Olivares et al. [55] applied plant growth-promoting bacteria to different crop fields and found an increase in maize grain production by 65% and tomato fruit production by 87.1%. Total yield and its components in this study were directly influenced by the number of split applications of N and P fertilizers. Tuber specific gravity controls the final price of the product as is an important quality parameter [56]. The current research revealed that with an increasing number of N and P fertilizer applications, values of specific gravity decreased. The results of the current study correlate with previous reports [56, 57]. Our research demonstrates a relationship between potato yield and balanced application of PGPB coated N and P biofertilizers which indicates MFA as an effective approach to enhance potato production, with reduced water pollution.

The substantial increase in N uptake of plants grown under MFA approach compared to those grown under CFA is a clear indication that optimum potato production is attaiable under MFA approach. This has wide-ranging advantages, including reduced environmental pollution associated with the loss of N and P into surface water bodies [58, 59]. These results align with the findings of Nyiraneza et al. [60] and Zebarth et al. [61], both of whom noted that variations in P uptake occur due to variations in applied rates and frequencies of fertilizers.

## Conclusion

To the best of our knowledge, this is the first ever report in Pakistan determining the application rate and frequency of PGPB coated N and P nutrients for improved growth, fluorescence, gas exchange, yield, and nutrient uptake of potato cv. Barna under semiarid and saline conditions of Cholistan. The modified fertilizer application (MFA) approach, encompassing the appropriate rate and frequency of PGPB-coated biofertilizers, represents a groundbreaking solution for potato cultivation in Cholistan, Pakistan. It significantly improves crop growth, yield, and quality while mitigating challenges posed by poor soils and environmental conditions, offering a sustainable approach for small-scale growers.

### Methodology

#### Study site

A field experiment was conducted at Horticulture Experimental Area (29°22′17.4″ N 71°45′53.6″ E), Department of Horticultural Sciences, The Islamia University of Bahawalpur, Pakistan during 2021–2022. This area is located in the Cholistan desert with a subtropical climate. Cholistan is a very hot desert in Pakistan. There is a wide range of 100–200 mm in the annual average rainfall. The monsoon (July–September) and the winter/spring (January–March) are the most common times for precipitation. The minimum and maximum average temperatures are 20 °C and 40 °C, respectively. Month-wise means of air temperature and relative humidity as well as total rainfall during the crop period (2021-22) were determined during the experimental period. The soils of Cholistan are generally saline, alkaline, and gypsiferous. The underground water is brackish containing more than 900 ppm salts [62]. Before the trial, soil samples were taken from the experimental site and analyzed for their physical and chemical characteristics using methods of Ryan et al. [63]. Soil samples were taken from five different cores in the experimental site, at two depths (0–15 cm) and (16–30 cm) with an auger (15 cm high and 2 cm in diameter) before planting. Then, the collected soil from five random cores was mixed. Soil texture [64], electrical conductivity (EC) [65], pH [66], total nitrogen (N) [67], available phosphorus [68], organic matter [69] and available potassium (K) [70] were determined in both layers of soil. Similarly, irrigation water was also analyzed for its physical and chemical properties.

### Plant material

**Fig. 8:**
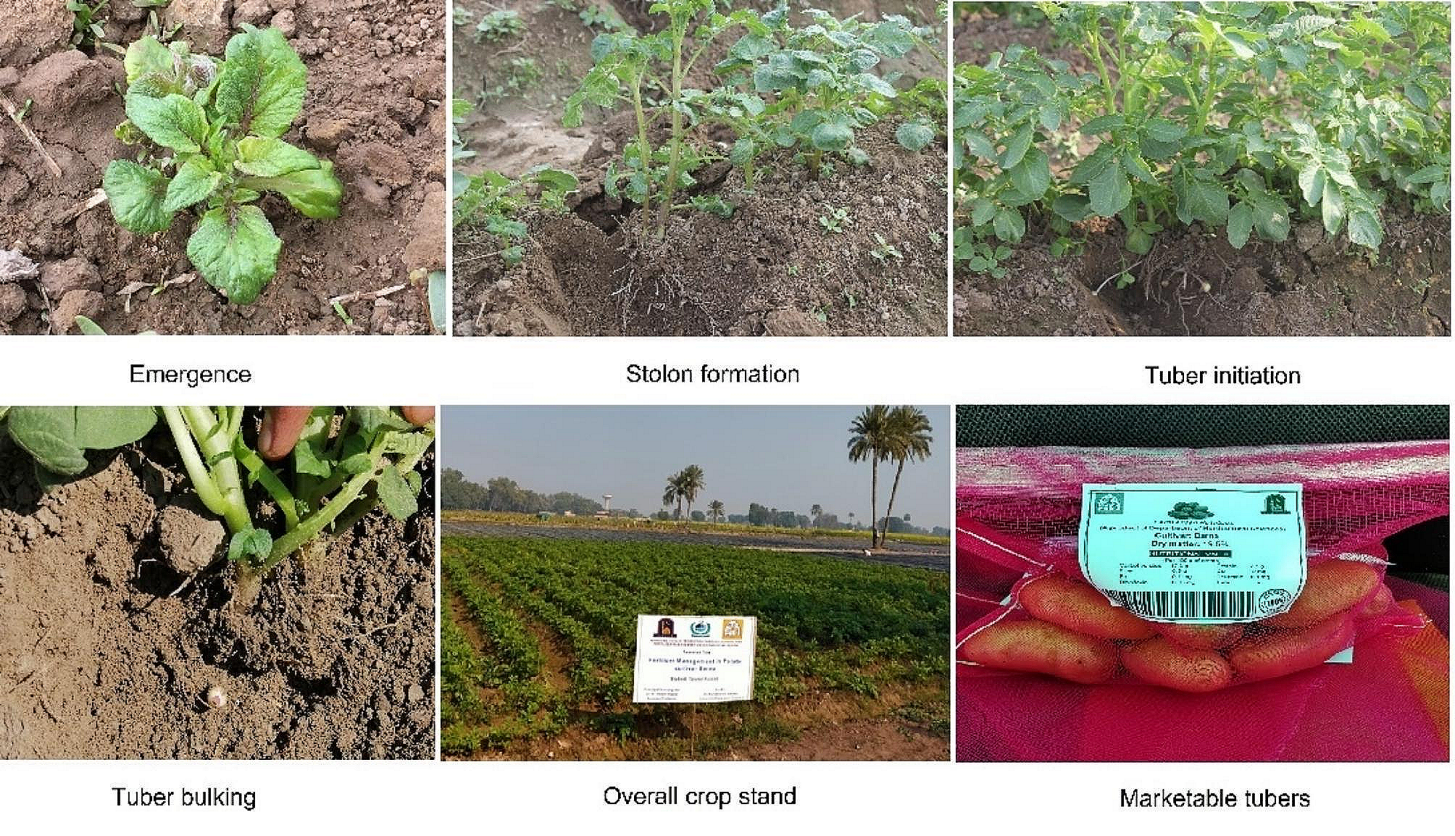
Different growth and development phases visually captured in potato cv. Barna during the experimental period

Potato cv. Barna, which exhibits taller height with upright stem and foliage structure, dark green leaves, late maturity, high yield, large round-oval tuber shape with red skin and pale-yellow flesh, and dry matter around 19.5% was used as planting material (Fig. 8).

### Fertilizer treatments

In the current experiment, two different treatments were set up, i.e., the conventional fertilizer application (CFA) approach, which was also kept as control and included traditional practices of (PGPB enriched) nitrogen (N) and phosphorus (P) (100 kg:75 kg acre^–1^) application in four splits (the doses of both N and P used in this treatment, were recommended by the Punjab Agriculture Department, Pakistan); and the alternate treatment was the modified fertilizer application (MFA) approach encompassing PGPB enriched N and P (80 kg:40 kg acre^–1^) application in nine splits. The rates were optimized using initial testing with N: P levels of 90:45, 80:40, and 70:35 kg acre^–1^. The number of splits in this trial were based according to total irrigations number. In both treatments, N and P were applied in the form of PGPB-coated diammonium phosphate (DAP) and urea (containing PGPB at the rate of 103 g^–1^) commercially available as Nutraful manufactured by Jaffer Agro Services Private Ltd. Schedules followed for fertilizer application under CFA and MFA approaches are given in Tables 3 and 4, respectively.

**Table 3:** Fertilizer (N and P containing) application schedule used for Conventional Fertilizer Application Approach

No.	Time of *N* and *P* application	Amount of fertilizer acre^–1^	Date
1	Time of land preparation	150 kg DAP	Oct 14, 2021
2	Third irrigation	50 kg urea	Nov 10, 2021
3	Fourth irrigation	50 kg urea	Nov 20, 2021
4	60 days after sowing	50 kg urea	Dec 12, 2021

DAP = diammonium phosphate

**Table 4:** Fertilizer (N and P containing) application schedule used for Modified Fertilizer Application Approach. DAP = diammonium phosphate

No.	Time of *N* and *P* application	Amount of fertilizer acre^–1^	Date
1	Time of land preparation	40 kg DAP	Oct 14, 2021
2	Second irrigation	23 kg DAP	Oct 27, 2021
3	Third irrigation	25 kg urea	Nov 10, 2021
4	Fourth irrigation	23 kg DAP	Nov 20, 2021
5	Fifth irrigation	20 kg urea	Dec 8, 2021
6	Sixth irrigation	20 kg urea	Dec 23, 2021
7	Seventh irrigation	25 kg urea	January 6, 2022
8	Eighth irrigation	25 kg urea	January 17, 2022
9	Ninth irrigation	25 kg urea	January 31, 2022

DAP = diammonium phosphate

### Experimental protocol

Healthy, medium-sized seed tubers (Mean weight: 75 ± 5 g) of cv. ‘Barna’ were planted manually by hands approximately 15 cm apart and 0.1 m deep in the ridges developed by a tractor-drawn ridger with a row-row spacing of 75 cm, on the 20th of October, 2021 and tubers were harvested 126 days after sowing, on February 17, 2022, using a spade. Each treatment was replicated four times and each replication unit had an area of 144 ft^2^. The trial was set up under a randomized complete block design.

### Field and laboratory measurements

The data related to potato growth, fluorescence, gas exchange, and nutrient uptake were recorded for five randomly selected potato plants, while total yield and its grade-based fractions were taken from the whole experimental unit.

### The growth-related attributes

The growth-related attributes such as plant height (cm), number of stems, and leaves plant^–1^, leaf area index, and leaf area index were recorded on the 70th day of planting. Leaf area was measured using the below formula developed by Firman and Allen [71]. Leaf area index was calculated by dividing the projected area of leaves over a unit of land.

1 $$\text{L}\text{o}\text{g}10 \left(\text{l}\text{e}\text{a}\text{f} \text{a}\text{r}\text{e}\text{a} \text{i}\text{n} \text{c}\text{m}2\right)=2.06\times \text{L}\text{o}\text{g}10\left(\text{l}\text{e}\text{a}\text{f} \text{l}\text{e}\text{n}\text{g}\text{t}\text{h} \text{i}\text{n} \text{c}\text{m}\right)-0.458$$

### The fluorescence-related attributes

The fluorescence-related attributes i.e., relative chlorophyll content, quantum yield of photosystem II (Φ_II_), non-photochemical quenching (Φ_NPQ_), non-regulatory energy dissipation (Φ_NO_), photosynthetically active radiations (PAR), magnitude of electrochromic shift (ECSt), proton flux (vH^+^), proton conductivity (gH^+^), linear electron flow (LEF), and leaf thickness (µm) in potato were also recorded on the 70th day of planting using a MultispeQ-Beta instrument and PhotosynQ platform software [72].

### The gas exchange-related attributes

The gas exchange-related attributes i.e., photosynthetic (µmol CO_2_ m^–2^ s^–1^) and transpiration (mmol H_2_O m^–2^ s^–1^) rates, stomatal conductance (mol H_2_O m^–2^ s^–1^) and intercellular CO_2_ (µmol CO_2_ mol^–1^) were determined using an infra-red gas analyzer (IRGA) (LCi-SD, ADC Bio-scientific, England) by selecting three fully developed healthy leaves from each plant (five plants in each replication). IRGA was monitored around solar midday at a light intensity of 850 to 1050 µmol m^–2^ s^–1^, leaf surface area of 6.25 cm^2^, the CO_2_ concentration of 390.12 µmol^–1^, the temperature of leaf surface (31.7–36.5 °C), airflow rate per unit area of leaf (U) 200.9 µmol s^–1^, atmospheric pressure (P) of 991 mBar and H_2_O partial pressure was 13.4 mBar. Water use efficiency was derived from the following formula as previously used by Haider [73].

2 $$Water use efficiency \left(WUE\right)=\frac{Photosynthetic rate \left({P}_{\text{N}}\right)}{Transpiration rate \left(E\right)}$$

### Yield related attributes

The yield-related parameters i.e., total, marketable (> 90 g), seed-grade (50–90 g), and goli (< 50 g) yields were determined by weighing tubers on an electronic scale. The damaged, diseased, and deshaped or deformed tubers were assumed downgrade. The tuber yield per treatment plot (kg 144 ft^–2^) was converted into (ton acre^–1^) using the following formula:

3 $$Tuber yield (ton/acre)=\frac{Weight of tubers per plot \left(kg\right) }{Area of the plot \left(144 ft2\right)}\times Area of an acre$$

Tuber diameter was recorded using Vernier Caliper (IP67, BEAPO Hardware Industrial Company, China) and specific gravity was determined from the following formula.

4 $$Specific gravity=\frac{Weight in air}{Weight in air-Weight in water}$$

### Nutrient uptake

Five plants were chosen at random from the harvesting area, and their haulm biomass was harvested, measured, and weighed. Additionally, ten tubers were selected from the harvested and weighed before being chopped into 10 mm wide strips. Weights were taken before and after oven drying subsamples of 500 g of each haulm biomass and tubers at 70 °C for 72 h. The samples were then ground with mortar and pestle for nutrient (N and P) analysis. The quantification of nitrogen was carried out using the Kjeldahl method, as outlined by Bremner [67]. On the other hand, phosphorus was analyzed using colorimetric analysis using a UV-vis spectrophotometer, following the procedure published by Murphy and Riley [68]. The determination of N and P nutrient uptake for haulms and tubers involved calculating the product of the dry weight of the tissues and the concentration of nutrients. The total nutrient uptake of the plant was obtained by summing the values for both tubers and haulms.

5 $$\begin{array}{l}{\rm{Plant Nutrient uptake = Haulm nutrient uptake}}\\\,\,\,\,\,\,\,{\rm{ + Tuber nutrient uptake}}\end{array}$$

To determine nutrient uptake efficiency of potato, the ratio of total potato nutrient uptake to nutrient supply was calculated by employing the below formula.

6 $$Nutrient uptake efficiency=\frac{Total plant nutrient uptake}{Nutrient supply}$$

### Data analysis

Data processing was carried out on Microsoft Excel 2016. The data were assessed by analysis of variance (ANOVA) using Statistix 9® for Windows (Analytical Software, Tallahassee, USA), and mean values were compared with the Tukey’s HSD Test at *P* < 0.05.

## Data Availability

All the data related to this work can be sourced from the corresponding authors.

## Abbreviations

ANOVA: Analysis of variance
CFA: Conventional fertilizer application
MFA: Modified fertilizer application
*C*_i_: Sub-stomatal CO_2_ level
*E*: Transpiration rate
gH^+^: Proton conductivity
*g*_s_: Stomatal conductance
IUB: The Islamia University of Bahawalpur
LEF: Linear electron flow
PARs: Photosynthetically active radiations
*P*_N_: Photosynthetic rate
SPAD: Soil and plant analysis development
SSC: Soluble solid content
Tukey’s HSD test: Tukey’s honestly significant difference test
vH^+^: Proton flux
WUE: Water use efficiency
Φ_II_: Quantum yield of photosystem II
Φ_NO_: Non-regulatory energy dissipation
Φ_NPQ_: Non-photochemical quenching
